# A Synthetic Derivative SH 66 of Homoisoflavonoid from Liliaceae Exhibits Anti-Neuroinflammatory Activity against LPS-Induced Microglial Cells

**DOI:** 10.3390/molecules29133037

**Published:** 2024-06-26

**Authors:** Md Samsuzzaman, Lalita Subedi, Seong-Min Hong, Sanha Lee, Bhakta Prasad Gaire, Eun-Ji Ko, Ji-Woong Choi, Seung-Yong Seo, Sun-Yeou Kim

**Affiliations:** 1 College of Pharmacy and Gachon Institute of Pharmaceutical Sciences, Gachon University, Incheon 21936, Republic of Korea; samsuzzaman238@gmail.com (M.S.); subedilali@gmail.com (L.S.); hongsm0517@gmail.com (S.-M.H.); sanha030@gmail.com (S.L.); samarpanbp@gmail.com (B.P.G.); imko1004@gachon.ac.kr (E.-J.K.); pharmchoi@gachon.ac.kr (J.-W.C.); 2 Department of Biochemistry and Molecular Biology, University of Maryland, Baltimore, MD 21201, USA

**Keywords:** homoisoflavonoid (SH66), microglia, proinflammatory cytokines, NLRP3 inflammasome, MAPK signaling, neuroinflammation

## Abstract

Naturally occurring homoisoflavonoids isolated from some Liliaceae plants have been reported to have diverse biological activities (e.g., antioxidant, anti-inflammatory, and anti-angiogenic effects). The exact mechanism by which homoisoflavonones exert anti-neuroinflammatory effects against activated microglia-induced inflammatory cascades has not been well studied. Here, we aimed to explore the mechanism of homoisoflavonoid SH66 having a potential anti-inflammatory effect in lipopolysaccharide (LPS)-primed BV2 murine microglial cells. Microglia cells were pre-treated with SH66 followed by LPS (100 ng/mL) activation. SH66 treatment attenuated the production of inflammatory mediators, including nitric oxide and proinflammatory cytokines, by down-regulating mitogen-activated protein kinase signaling in LPS-activated microglia. The SH66-mediated inhibition of the nucleotide-binding oligomerization domain-like receptor family pyrin domain containing 3 (NLRP3) inflammasome complex and the respective inflammatory biomarker-like active interleukin (IL)-1β were noted to be one of the key pathways of the anti-inflammatory effect. In addition, SH66 increased the neurite length in the N2a neuronal cell and the level of nerve growth factor in the C6 astrocyte cell. Our results demonstrated the anti-neuroinflammatory effect of SH66 against LPS-activated microglia-mediated inflammatory events by down-regulating the NLRP3 inflammasome complex, with respect to its neuroprotective effect. SH66 could be an interesting candidate for further research and development regarding prophylactics and therapeutics for inflammation-mediated neurological complications.

## 1. Introduction

Phytomedicines are reported to have beneficial effects over conventional medication, especially for neurological complications, and are gaining interest for their anti-neuroinflammatory potential [1]. Among these phytomedicines, homoisoflavonoids are an important naturally occurring phytochemical group isolated from the Asparagaceae, Fabaceae, Liliaceae, Polygonaceae, Orchidaceae, and Gentianaceae families, and contain 16-carbon skeleton structures with one or more carbon constituents than flavonoids and isoflavonoids [2]. Homoisoflavonoids are reported to have diverse biological activities including antioxidant, anti-inflammatory, and anti-angiogenic activities [3]. Homoisoflavonoids are categorized into five structural types: sappanin-type, scillascillin-type, brazilin-type, caesalpin-type, and protosappanin-type [4]. Among them, sappanin-type homoisoflavonoids are the most isolated and studied, and mainly consist of 3-benylchromanone and 3-benzylidenechromanone structures. The chemical libraries for these have been developed by our group and evaluated against chronic neuroinflammation induced by activated microglia [5].

**Figure 1:**
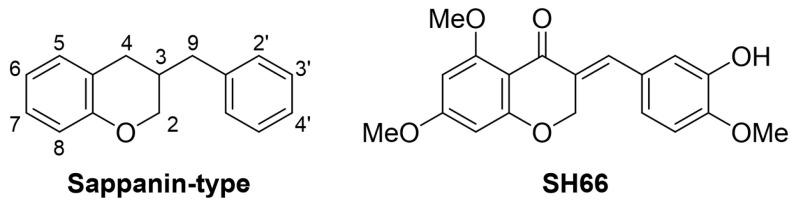
Chemical structure of saponins-type homoisoflavonoid and SH66.

Herein, we describe a homoisoflavonoid-based anti-neuroinflammatory effect against toll-like receptor (TLR)-mediated inflammation in microglial cells. Through screening our in-house homoisoflavonoid-based chemical library, we identified a potent anti-neuroinflammatory agent, (E)-3-(3-hydroxy-4-methoxybenzylidene)-5,7-dimethoxychroman-4-one (named SH66) (see Figure 1). To date, SH66 has not yet been studied for its biological activity, except for our study related to its anti-angiogenic activity against ocular neovascularization, although it was mentioned in a synthetic study in which 3-benzylidenechromanones such as SH66 were converted into 3-benzylchromone through base-induced rearrangement [5]. Interestingly, Wang et al. developed homoisoflavonoid derivatives as potent dual inhibitors of monoamine oxidase B and cholinesterase for Alzheimer’s disease (AD) [6].

It is well known that neuroinflammation and neurodegeneration are critical pathological events in almost all types of neurological complications [e.g., AD, Parkinson’s disease (PD), amyotrophic lateral sclerosis (ALS), and multiple sclerosis (MS)] [7,8]. Neuroinflammation can initiate and aggravate neurodegeneration via axonal degeneration or apoptosis, or by the overactivation of endogenous or exogenous immune cells [9]. Additionally, it was reported that the neuroinflammation induced by the activation of microglia might be central events leading to neurodegeneration [10].

Microglia as a resident macrophage regulate the immune response in the central nervous system and induce the inflammation process in response to brain damage [11]. The activated microglia stimulate the inflammatory cytokines (i.e., interleukin (IL)-1β, IL-6, tumor necrosis factor-α (TNF-α), which contributes to neuronal death [12]. Specifically, toll-like receptor 4 (TLR4) is an important receptor that mediates the inflammatory pathways and produces related cytokines [13]. It already reported that TLR4 perceives with amyloid beta that contributor to AD via activated microglia [14]. Especially, mitogen-activated protein kinases (MAPKs) trigger the nuclear factor-κB (NF-κB) and produce the inducible nitric oxide synthase (iNOS) and cyclooxygenase-2 (COX-2) via TRP4-mediated inflammatory signaling [15]. Thus, it suggests that targeting TRL4 signaling is a strategy for finding AD therapeutic treatment.

In addition, inflammasome formation and the excessive production of pro-inflammatory cytokines by activated microglia are the major reason for the initiation and severity of neuroinflammation [16]. The nucleotide-binding oligomerization domain-like receptor family pyrin domain containing 3 (NLRP3) is a sensor for inflammasome-mediated adverse effects [17]. The activation of NLRP3 forms the NLRP3 inflammasome complex containing caspase-activation and recruitment domain (ASC) and pro-caspase-1, which contributes to cellular and tissue injury by the excessive production of pro-inflammatory cytokine interleukin 1β (IL-1β) [18]. An increase in NLRP3 and the activation of the NLRP3 inflammasome complex in activated microglia during neuroinflammation is one of the key pathways leading to neurodegeneration in diverse neuroinflammatory disorders, including ischemic injury [16]. Controlling these cascades can lower neuroinflammation and subsequent neurodegeneration. Thus, regulating the NLRP3 inflammasome is considered a therapeutic target for treating neurodegenerative diseases as AD.

Several phytochemical-based synthetic compounds have been reported to control these events, and these might ultimately support the development of prophylactics and therapeutics for neurological ailments. Homoisoflavonoids have also been reported to lower the production of inflammatory mediators in lipopolysaccharide (LPS)-activated microglia, however their exact mechanism has not been investigated thoroughly yet [19,20]. In the present study, we evaluated the anti-inflammatory mechanisms of homoisoflavone SH66 against activated microglia-induced inflammatory cascades in LPS-stimulated BV2 microglial cells.

## 2. Results

### 2.1. SH66 Synthesis

**Scheme 1:**
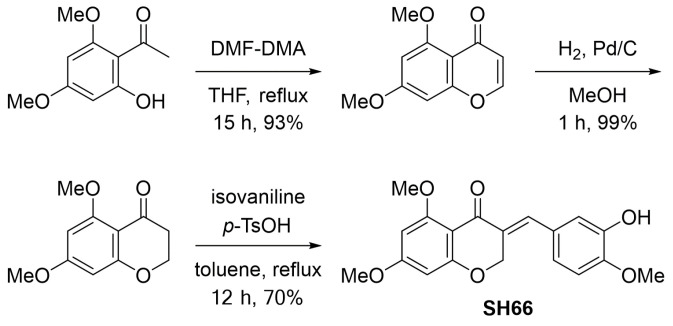
Synthesis of SH66.

Considerable attention has been paid to the unique structures and diverse biological activities of homoisoflavonoids, including the antioxidant, anti-inflammatory, and anti-angiogenic activities [4]. Homoisoflavonoids are structurally categorized as sappanins, scillascillins, brazilins, caesalpins, or protosappanins [3,4]. Among them, sappanins that have a chroman-4-one skeleton with a 3-benzyl or 3-benzylidene moiety have been mostly isolated from nature, and many synthetic and biological studies have been reported [21]. We recently developed a naturally occurring homoisoflavonoid, cremastranone, and its synthetic derivatives that have potent anti-angiogenic activity. While 5,6,7-trioxygenated homoisoflavonoids have been mainly developed as anti-angiogenic agents, the present study was performed based on the potential of a homoisoflavonoid with a 5,7-dimethoxy such as SH66 for anti-neuroinflammatory activity. SH66 (**1**) consists of a 3-benzylidenechroman-4-one skeleton with 5,7-dimethoxy in the A ring and 3-hydroxy-4-methoxy in the B ring (Figure 1). Through efforts to optimize the synthesis of homoisoflavonoids established by us [5], SH66 was synthesized in a total yield of 64% by a three-step process using 2′-hydroxy-4′,6′-dimethoxyacetophenone (**2**) as a starting material, which was treated with N,N-dimethylformamide dimethyl acetal (DMF-DMA), followed by the catalytic hydrogenation of the resulting 5,7-dimethoxy-4H-chromen-4-one (**3**) to give 5,7-dimethoxychroman-4-one (**4**) (Scheme 1). Finally, SH66 was obtained via aldol condensation of 5,7-dimethoxychroman-4-one (4) and isovanillin using catalytic p-toluenesulfonic acid (p-TsOH).

### 2.2. SH66 Lowers Nitric Oxide (NO) Production and Protein Levels of iNOS and COX-2 in TLR-Activated BV2 Microglia Cells

**Figure 2:**
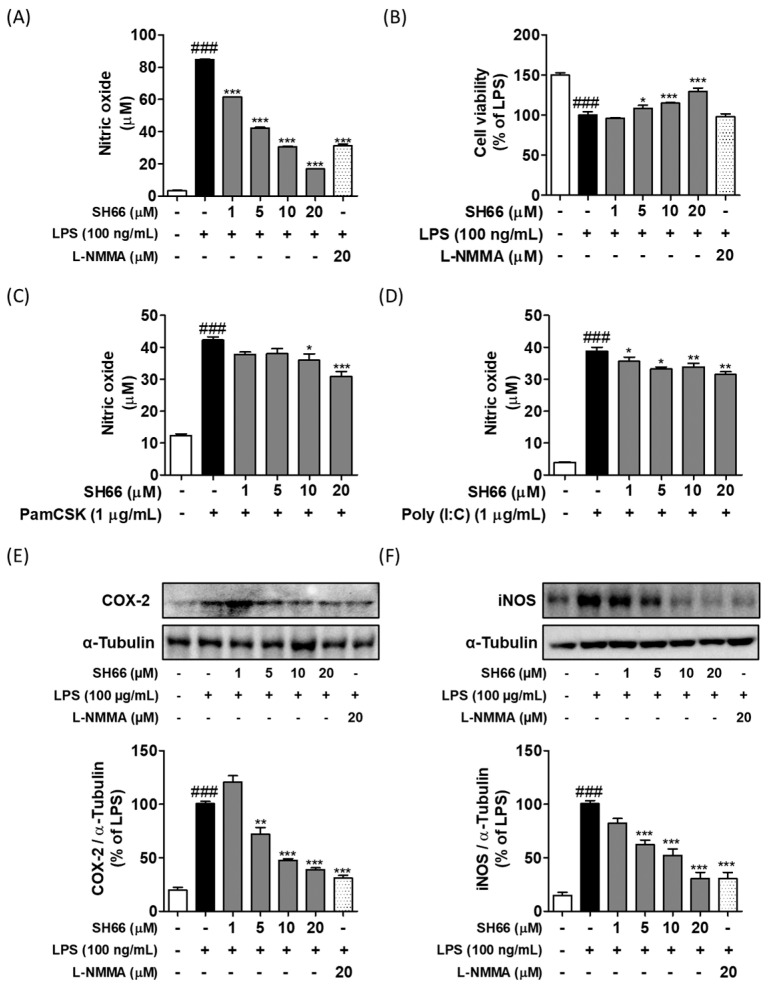
SH66 inhibits the nitric oxide (NO) production and expression of iNOS and COX-2 in TLR-activated microglia. SH66 was pre-treated for 30 min in BV2 microglial cells followed by the treatment of TLR (1–4) agonists and incubated for 24 h. TLR’s agonist, like LPS, PamCSK, and Poly (I:C), was used for the activation of TLR (1–4). Cells were incubated overnight after the activation of microglia with agonists, the conditioned medium of the overnight incubated cells was used to evaluate the amount of NO, and attached cells were analyzed with MTT reagent for cell viability assay. (**A**–**D**) NO production and cell viability. SH66 was pre-treated and followed by LPS activation for 6 h and cells were harvested for western blot analysis. (**E**,**F**) Protein levels and their densitometric analysis for COX-2 and iNOS. α-tubulin was used as loading control. The data shown represent the mean ± SEM (*n* = 3). * *p* < 0.05, ** *p* < 0.01, and *** *p* < 0.001 vs. LPS alone. ### *p* < 0.001 vs. untreated control group.

Microglial cells were treated with SH66 following LPS activation. Only LPS-treated cells dramatically increased NO production, and this level was diminished in a concentration-dependent manner in the group treated with different concentrations of SH66. The inhibition of NO following treatment with 10 μM of SH66 showed almost similar potency to that of the positive control (20 μM), that is, N^G^-Monomethyl-L-arginine acetate (L-NMMA) as a nitric oxide synthase inhibitor (Figure 2A). LPS showed significant toxicity to microglial cells, and this was recovered in the SH66-treated group (5–20 μM; Figure 2B). As LPS is a TLR4 agonist and SH66 works against LPS-treated microglia, we further tested the effect of SH66 against other TLR agonists. Treatment with the TLR1,2 agonist synthetic triacylated lipopeptide (pamCSK, 1 μg/mL) and the TLR3 agonist polyinosinic:polycytidylic acid [Poly(I:C), 1 μg/mL] also significantly increased NO production, and SH66 lowered this effect at 10 and 20 μM against PamCSK and from 1 to 20 μM against Poly(I:C) (Figure 2C,D). Together, these results suggest that the inhibition of inflammatory NO production by activated microglia following SH66 pre-treatment is not selective only for TLR4 but also for other TLR ligands, resulting in the multifunctional potential of SH66. Since the effect of SH66 against LPS-activated cells was striking, we selected the LPS model for further research. We evaluated the protein levels of iNOS and COX-2 in LPS-activated microglia. As expected, pre-treatment with SH66 dramatically lowered the levels of iNOS and COX-2 in LPS-activated microglial cells (Figure 2E,F). The inhibition of iNOS might be the reason for the notable inhibition of SH66 in LPS-activated microglia.

### 2.3. SH66 Controls MAPK-Mediated Effector Signaling to Inhibit Inflammatory Cascades in LPS-Activated BV2 Microglia Cells

**Figure 3:**
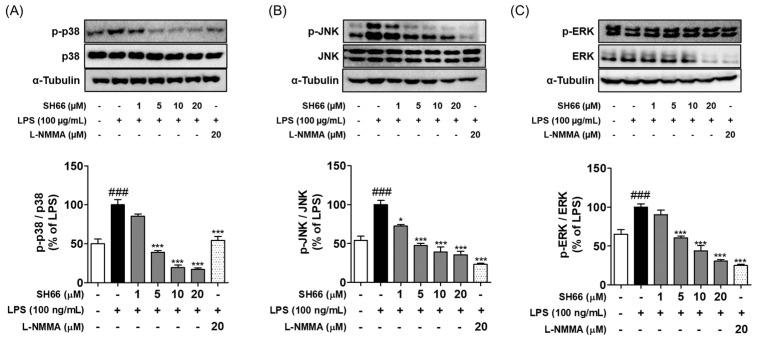
SH66 inhibits MAPK phosphorylation in LPS-primed microglial cells. BV2 cells were pre-treated with SH66 (1–20 μM) followed by the priming of LPS (100 ng/mL) and incubated for 30 min. Protein level was analyzed by western blot analysis. (**A**–**C**) Protein levels and their quantitative analysis for p-p38/p38, p-JNK/JNK, and p-ERK/ERK. α-tubulin was used as loading control. The data shown represent the mean ± SEM (*n* = 3). * *p* < 0.05 and *** *p* < 0.001 vs. LPS alone. ### *p* < 0.001 vs. untreated control group.

MAPK signaling plays a key role in modulating inflammation in activated microglia through the TLR4 pathway [22]. MAPK signaling mediates the initiation of several inflammatory cascades, including inflammasome formation and the induction of transcription and translation of pro-inflammatory mediators. Thus, we further examined the protein levels of MAPK proteins following SH66 pre-treatment and LPS activation. LPS activation significantly increased the phosphorylation of MAPK proteins, while SH66 pre-treatment significantly reversed the phosphorylation of all targets, including p38, extracellular signal-regulated kinase (ERK), and c-Jun N-terminal kinase (JNK) (Figure 3A–C). Regarding the pattern of MAPK phosphorylation inhibition, p38 and ERK inhibition seemed to occur in a concentration-dependent manner. JNK phosphorylation inhibition by SH66 seemed to be almost the same at all treatment concentrations.

### 2.4. SH66 Treatment Inhibited the NLRP3 Inflammasome Complex and Subsequent IL-1β Activation in LPS-Activated BV2 Microglia Cells

**Figure 4:**
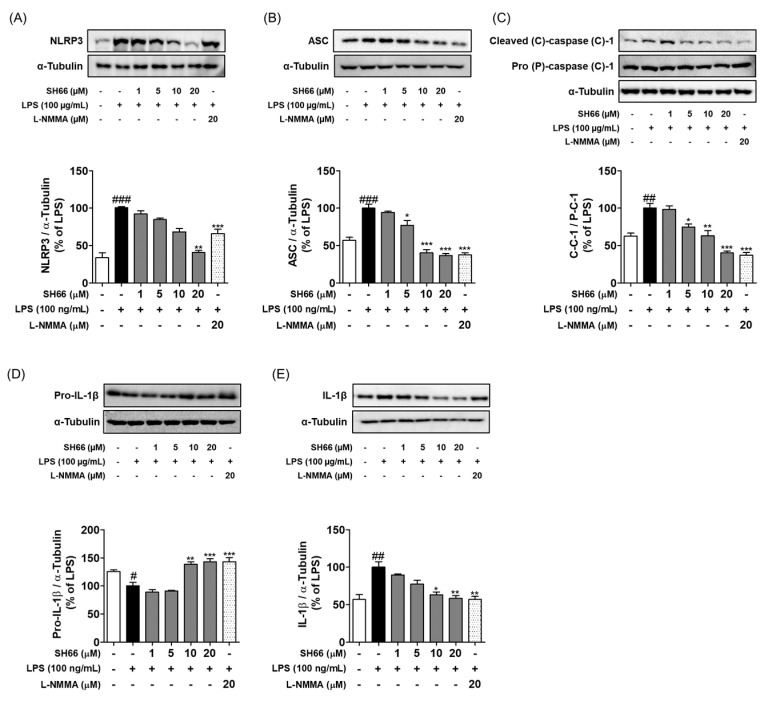
SH66 inhibits NLRP3 inflammasome induction and activation in LPS-primed microglial cells. BV2 cells were pre-treated with SH66 (1–20 μM) followed by the priming of LPS (100 ng/mL) and incubated for 6 h. Protein level was analyzed by Western blot analysis. (**A**–**E**) Protein levels and their quantitative analysis for NLRP3, ASC, pro-caspase-1, cleaved-caspase-1, pro- IL-1β, and IL-1β. α-tubulin was used as loading control. The data shown represent the mean ± SEM (*n* = 3). * *p* < 0.05, ** *p* < 0.01, and *** *p* < 0.001 vs. LPS alone. # *p* < 0.05, ## *p* < 0.01, and ### *p* < 0.001 vs. untreated control group.

The activation of the inflammasome pathway is a major event that leads to neuroinflammation [18]. MAPKs are key effector signaling molecules that activate NLRP3 to form the inflammasome complex in the presence of ASC and caspase 1 [17]. This inflammasome is responsible for the conversion of IL-1β to mature IL-1β [23]. These events are responsible for the tremendous secondary damage to neuronal cell types and the CNS environment. Supporting this fact, we also observed that the LPS-treated group showed significantly higher levels of NLRP3-ASC-cleaved caspase-1, followed by the increased conversion of pro-IL-1β to mature IL-1β (Figure 4A–E). These changes were reversed in the SH66 pre-treated group in a concentration-dependent manner.

### 2.5. SH66 Treatment Inhibited the Production of Pro-Inflammatory Cytokines in LPS-Activated BV2 Microglia Cells

**Figure 5:**
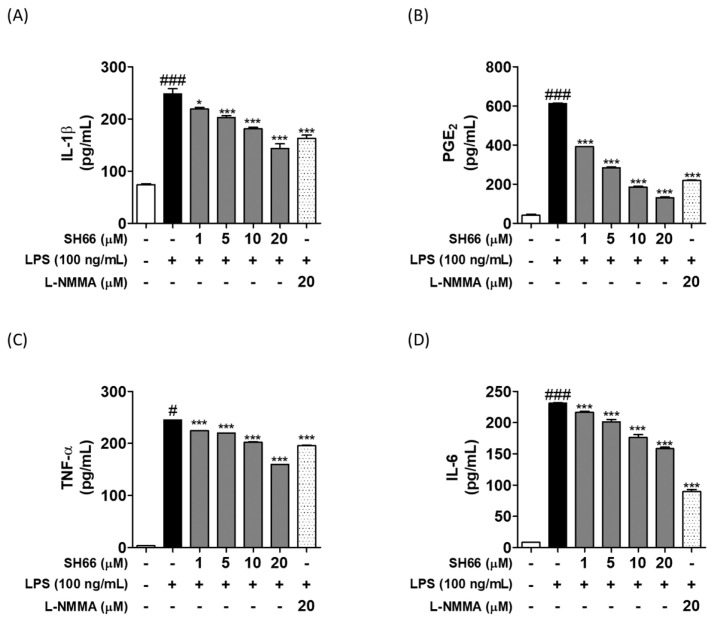
SH66 inhibits the production of inflammatory mediators and pro-inflammatory cytokines in LPS-primed microglia. SH66 was pre-treated in microglial cells followed by the priming of LPS (100 ng/mL) and incubated overnight. Conditioned medium from the overnight incubated cells was used to measure the secreted level of inflammatory mediators and pro-inflammatory cytokines. (**A**–**D**) Secreted level of IL-1β, PGE2, TNF-α, and IL-6 in LPS-primed microglia. The data shown represent the mean ± SEM (*n* = 3). * *p* < 0.05 and *** *p* < 0.001 vs. LPS alone. # *p* < 0.05 and ### *p* < 0.001 vs. untreated control group.

The inhibition of p38, JNK, and ERK phosphorylation signaling was further confirmed by the inhibition of proinflammatory cytokine levels under the same treatment conditions. The inhibition of IL-1β and prostaglandin E2 (PGE2) (Figure 5A,B) was more significant and dramatic. The inhibition of TNF-α, IL-1β, and PGE2 at 20 μM of SH66 pre-treatment was better than that of L-NMMA; however, it was not more potent than L-NMMA in inhibiting IL-6 production (Figure 5A–D).

### 2.6. SH66 Treatment Induced Neuroprotection by Increasing the Level of Nerve Growth Factor (NGF) and Neurite Outgrowth, and Decreased Activated Microglia-Mediated Neuronal Death

**Figure 6:**
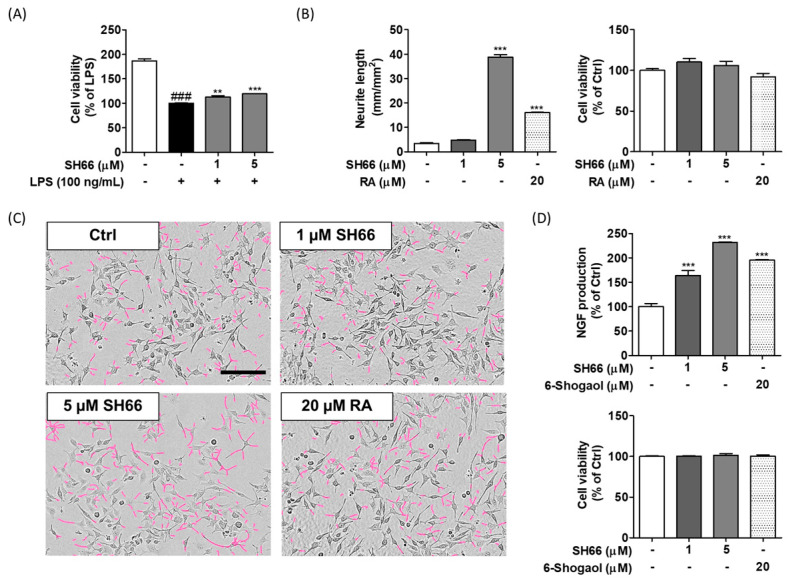
SH66 shows neuroprotection in central nervous system (CNS) cell lines. (**A**) Microglial cells were pre-treated with SH66 followed by LPS-priming and incubated for overnight. Conditioned medium (CM) from the plates was transferred to the seeded N2a neuronal cells to see the LPS-mediated toxicity and the role of SH66 in protection or toxicity. Cell viability of N2a cells treated with CM from activated microglia. The cell viability in N2a cells measured by MTT assay. (**B**,**C**) Neurite length of N2a cells were measured by using IncuCyte imaging system. N2a neuronal cells were treated with SH66 and retinoic acid (RA, positive control). The cell viability in N2a cells measured by MTT assay. (**D**) Secreted level of NGF production measured by ELISA assay. N2a neuronal cells were treated with SH66 and 6-shogaol (positive control) and cell viability in C6 astrocyte measured by MTT assay. The data shown represent the mean ± SEM (*n* = 3). ** *p* < 0.01 and *** *p* < 0.001 vs. LPS alone. ### *p* < 0.001 vs. untreated control group.

SH66 induced cell survival against activated microglia-induced neuronal toxicity, as evidenced by cell viability (Figure 6A). Treating neuronal N2a cells with SH66 increased neurite levels, especially at 5 μM (Figure 6C) without cellular toxicity in the direct treatment (Figure 6B). The induction of the nerve growth factor by astrocytes is believed to be neuroprotective against several types of neurotoxicity. Treating astrocytes with SH66 resulted in a significant induction of NGF production without any cellular toxicity, suggesting a positive effect of inducing neuroprotection (Figure 6D). The induction of NGF is also known to be related to increased neurite outgrowth, and we also observed a similar effect.

## 3. Discussion

Homoisoflavonoids are mainly obtained from the Asparagaceae, Fabaceae, and Liliaceae families of medicinal plants [2]. They appear to be structurally similar to flavonoids, but they have one or more carbons in comparison to flavonoids [2]. Because they are uncommon, few biological and medicinal studies have been reported thus far [5]. To date, only plants containing homoisoflavonids have been used for treating neurological diseases in traditional medicine [4]. The suggested effects of these homoisoflavonoids include antioxidant, anti-inflammatory, immunomodulatory, anti-angiogenic, anti-diabetic, vasorelaxant, and anti-microbial effects. The exact mechanism by which homoisoflavonoids down-regulate neuroinflammatory events has not yet been elucidated. Being naturally available phytochemicals with multiple pharmacological and therapeutic effects, homoisoflavonones have attracted interest for further research regarding their detailed mechanisms and potential for use in novel treatments.

The present study demonstrated the strong anti-neuroinflammatory potential of the homoisoflavonoid SH66 for the first time. SH66 significantly lowered the levels of inflammatory mediators and proinflammatory cytokines in LPS-activated microglial cells in an in vitro model of neuroinflammation. The SH66-mediated control of the NLRP3-inflammasome pathway is a key target responsible for its anti-neuroinflammatory effects. The LPS-mediated modulation of TLR4-MAPK-NLRP3-ASC-Caspase1-IL-1β in LPS-activated microglia was identified as the underlying mechanism of action of SH66. Moreover, SH66 showed the inhibitory effect of NO production as similar to isoflavonoid compounds derived from soybean (i.e., daidzin, daidzein, genistin, and genestein) (Figure S1, Supplementary Materials). Especially, homoisoflavonoid SH66 also showed good biological activity such as equol, which is a daidzein gut metabolite showing the neuroprotective effect (Figure S1, Supplementary Materials) [24]. It indicated that SH66 might be a potential therapeutic phytochemical for neuroprotection via regulating the NLRP3 inflammasome pathway.

TLRs are known to be involved in the pathogenesis of the majority of neurological complications either through the activation of the inflammasome or by NF-κB-mediated transcription and translation of inflammatory mediators in several diseases, including AD, traumatic brain injury, stroke, meningitis, spinal cord injury, and MS [25,26]. Based on these facts, the utilization of TLR antagonists and targeting the downstream mechanism could help researchers to identify potential candidates that can be utilized for preventive as well as therapeutic strategies against such conditions. In this study, we used LPS as a TLR4 agonist, Palm CSK as a TLR 1,2 agonist, and Poly(I:C) as a TLR3 agonist. SH66 inhibited TLR-mediated NO production non-selectively (Figure 1). The inhibition of NO in TLR4-mediated pathways seems to be more promising.

TLRs play a vital role in the expression of NLRP inflammasome proteins as well as IL-1β release via activated NF-κB and MAPK pathways, and these are well known as a modulator for the expression of the NLRP inflammasome and IL-1β level under inflammatory progression [27]. Indeed, many studies focused on the relationship between MAPKs and the NLRP3 inflammasome [27,28,29]. Thus, we further investigate the possible anti-neuroinflammatory mechanisms of SH 66 in LPS-induced microglial cells. LPS-TLR4-mediated NLRP3 inflammasome activation is regulated by two types of priming signals in microglia. Firstly, MAPK effector signaling and NF-κB have mediated the induction of the NLRP3 inflammasome complex. Secondly, the NRLP3 triggers the assembly of inflammasome via apoptosis-associated speck-like protein containing a caspase-recruitment domain (ASC) and effector protein pro-caspase-1,and increase the secretion of proinflammatory cytokines [30]. As SH66 inhibited the levels of inflammatory mediators such as NO, iNOS, and COX-2 in LPS-activated microglia (Figure 2), we further evaluated its role in controlling MAPKs. The significant inhibition of MAPK phosphorylation, especially p38 and ERK, was observed (Figure 3B,C). A previous study revealed that ERK and p38-mediated signaling are associated with NLRP3 inflammasome complex induction [4]. Our results also support this finding. The phosphorylation of JNK was also significant at all treatment concentrations, and this fact is supported by a previous study, in which the authors showed that the inhibition of JNK phosphorylation can also control NLRP3 inflammasome complex induction [31]. In this study, we suggested that JNK phosphorylation might be a critical player in the cleavage/activation of caspase 1 in NLRP3-inflammasome pathways that are dependent on ASC or NLRP3-ASC-caspase-1 assembly-mediated inflammasome complex-regulated NLRP3-inflammasome signals [31]. Based on these previous studies, p38 and ERK phosphorylation might be responsible for NLRP3 inflammasome complex induction. In addition, JNK phosphorylation determines NLRP3 inflammasome complex activation, ASC formation and activation, and caspase-1 activation, which ultimately results in the activation of matured proinflammatory IL-1β from pro-IL-1β (Figure 4).

It seems that the non-specific inhibition of MAPKs strongly down-regulates NLRP3 inflammasome induction/activation. We determined the protein levels of NLRP3, ASC, cleaved caspase-1, and IL-1β in LPS-activated microglia. As expected, LPS-mediated NLRP3 levels significantly lowered the protein levels of NLRP3 in a concentration-dependent manner, especially in the 10 and 20 μM of SH66 pre-treated groups (Figure 4A). At the same time, a sharp decline in the protein levels of ASC and cleaved-caspase 1, and ultimately reduced levels of mature IL-1β were observed (Figure 4B–E). Based on these results, we found that the inhibition of MAPKs by SH66 pre-treatment differentially regulated both NLRP3 inflammasome complex activation and priming signals.

We further determined the levels of other pro-inflammatory mediators and cytokines. The inhibition of IL-1β protein level was further clarified by the secreted level of IL-1β in the CM of LPS-activated microglia following SH66 treatment (Figure 5 and Figure 6). SH66 pre-treatment also significantly lowered the secreted levels of PGE2, TNF-α, and IL-6 (Figure 5). Altogether, the SH66-mediated inhibition of the NRLP3 inflammasome complex activation might lower the protein level and release of IL-1β, while the inhibition of other inflammatory mediators further supports the anti-inflammatory effect of SH66 against LPS-activated microglia (Figure 4 and Figure 5).

The inhibition of the secretion of inflammatory mediators in the conditioned medium (CM) of SH66 might be a possible reason for the significant protection of the neuronal cells in the microglial CM-treated N2a cell viability assay (Figure 6A). This result indicates that the SH66-mediated inhibition of inflammatory mediators and inflammasome pathway activation might lower activated microglia-mediated neurotoxicity. Neuronal cell survival or neuroprotection can be achieved through several mechanisms, among which the inhibition of neuroinflammation, systemic inflammation, and related inflammatory events or mediators is foremost [30]. Another route of neuroprotection is through the induction of neurite length, and this can be achieved by increasing the production of neurotrophins, including NGF in neurons and astrocytes [32]. SH66 showed increased neurite length in neuronal cells and at the same time increased NGF levels in astrocytes, further supporting the reason for neurite outgrowth induction.

**Figure 7:**
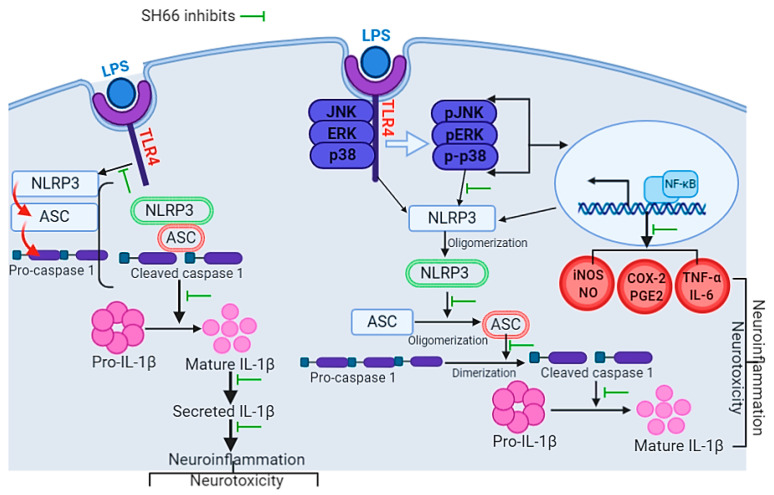
Schematic diagram of how SH66 targets NLRP3 to lower the level of inflammatory mediators and how it can participate in lowering neuroinflammation and neurotoxicity.

Taken together, homoisoflavonoid SH66 inhibited the level of NO against TLR1-, 2-, 3-, and 4-mediated downstream pathways in activated microglia with respective receptor agonists. SH66 inhibited NLRP3 inflammasome complex induction by lowering p38 and ERK phosphorylation, while inhibiting NLRP3 inflammasome complex activation by lowering JNK phosphorylation and inhibiting NLRP3-ASC-caspase 1 assembly, which subsequently lowered the levels of the inflammatory mediators in LPS-activated microglia. Interestingly, the inhibition of inflammatory mediators in the CM of SH66-pretreated microglia showed less toxicity to neuronal cells compared to LPS alone. The overall mechanism of SH66 to lower the inflammatory condition is summarized in Figure 7. Therefore, we suggest that SH66 may protect neuronal cells from microglial toxic responses. In addition, the upregulation of neurotrophins and neurite length in astrocytes and neuronal cells further supports the neuroprotective effect of SH66. Further research on SH66 in animal models might support SH66 as a possible candidate for the treatment of several neurological complications where activated microglia-derived inflammatory biomarkers and the NLRP3 inflammasome complex play critical roles.

## 4. Materials and Methods

### 4.1. Materials

Dulbecco’s modified Eagle’s medium, 3-(4,5-dimethylthiazol-2-yl)-2,5-diphenyltetrazolium bromide (MTT), fetal bovine serum, and penicillin–streptomycin (PS) were purchased from Invitrogen (Carlsbad, CA, USA). Lipopolysaccharide and L-NMMA were purchased from Sigma-Aldrich (St. Louis, MO, USA). Protein lysis buffer (Pro-Prep) was obtained from iNtRON Biotechnology (Seongnam-si, Gyeonggi-do, Republic of Korea). The primary antibodies against iNOS, COX-2, and α-tubulin were purchased from Santa Cruz Biotechnology (Dallas, TX, USA). Other primary antibodies such as α-tubulin, p38, JNK, ERK, pJNK, pp38, pERK, NLRP3, ASC, pro-caspase-1, cleaved caspase-1, pro-IL-1β, and IL-1β were purchased from Cell Signaling Technology (Beverly, MA, USA). Competitive enzyme-linked immunosorbent assay (ELISA) kits for PGE2, TNF-α, IL-6, and IL-1β were purchased from R&D Systems (Minneapolis, MN, USA). iNOS was purchased from Abcam (Cambridge, UK). The synthesis and characterization of SH66 were performed as described previously [5].

### 4.2. NO Production and Cell Viability Assays

BV2 microglial cells, originally developed at the University of Perugia (Perugia, Italy) by Dr. V. Bocchini, were kindly provided by Dr. E. Choi from Korea University, Seoul, Republic of Korea [33]. BV2 cells were maintained in DMEM and stored in a humidifier incubator at 37 °C and 5% CO_2_. BV2 cells at a density of 4 × 10^4^ cells/well were seeded in a 96-well plate and incubated overnight. Cells were pre-treated with SH66 or vehicle for 30 min followed by LPS (100 ng/mL) activation and incubated during 24 h. Nitric oxide (NO) production was measured in CM using the Griess reagent assay as described previously [24]. Briefly, 50 μL of CM from the treated cells was mixed with an equal volume of Griess solution, and the solution turned a pinkish color due to the presence of NO. The solution was quantified by measuring the absorbance at a wavelength of 540 nm using a microplate reader. The attached cells were used to measure the cytotoxicity via MTT assay as described previously. Briefly, cells were incubated with 100 μL of MTT solution (0.5 mg/mL) for approximately 1 h in a dark incubator. The reaction between MTT and the mitochondrial enzymes of live cells resulted in the blue staining of the live cells. The MTT solution was removed and 200 μL of dimethyl sulfoxide (DMSO) was added. This resulted in the production purple formazan that was used to quantify the live cells by measuring the absorbance at 570 nm using a microplate reader (Molecular Devices, San Jose, CA, USA).

### 4.3. Western Blot Analysis

Protein levels were analyzed by western blot analysis as described previously [24]. BV2 cells (1.5 × 10^6^ cells/well) were seeded into a six-well plate and pre-treated with SH66 in the presence or absence of LPS and incubated for different time periods. The cells were washed and lysed with Pro-Prep lysis buffer. Proteins (30 μg) were separated by sodium dodecyl sulfate-polyacrylamide gel electrophoresis (SDS-PAGE) to determine their expression. The separated proteins were blotted onto nitrocellulose membranes and incubated overnight with primary antibodies against iNOS, COX-2, p38, JNK, ERK, pJNK, pp38, pERK, NLRP3, ASC, pro-caspase-1, cleaved caspase-1, pro-IL-1β, IL-1β, and α-tubulin (1:1000). Protein bands were visualized using ECL reagent (Amersham Pharmacia Biotech, Little Chalfont, UK). The protein band intensity was quantified using Image Master™ 2D Elite software (version 3.1, Amersham Pharmacia Biotech, Little Chalfont, UK).

### 4.4. Preparation of Conditioned Medium (CM) from BV2 Microglia Cells

BV2 microglia cells were seeded (4 × 10^4^ cells/well) in a 96-well plate and incubated overnight. After 1 day, cells were pre-treated with SH66 or vehicle followed by LPS (100 ng/mL) activation and incubated overnight. The CM was collected and then treated to seeded N2a cells (2 × 10^4^ cells/well).

### 4.5. Neurite Outgrowth Assay

In the neurite outgrowth assay, N2a cells (American Type Culture Collection, Manassas, VA, USA) were seeded (1 × 10^5^ cells/well) onto 12-well plates and treated with SH66 for 24 h. The neurite outgrowth was measured by using the IncuCyte imaging system (Essen Instruments, Ann Arbor, MI, USA) from 0 h to 24 h of treatment in each 2 h interval.

### 4.6. ELISA Kit Assay

For measuring the levels of pro-inflammatory cytokine, BV2 cells were pre-treated with vehicle and different concentrations of SH66 following LPS activation, and then incubated for 24 h. CM from the treated cells was collected and stored at −80 °C until use. Stored CM was used to measure the secreted levels of inflammatory mediators and pro-inflammatory cytokines using commercial ELISA kits. The secreted levels of IL-1β, PGE_2_, TNF-α, and IL-6 were determined using the protocol provided by the manufacturer (R&D Systems).

For measuring the levels of NGF production, C6 cells (Korean Cell Line Bank, Seoul, Republic of Korea) were seeded (1 × 10^5^ cells/well) and treated with SH66 for 24 h. CM from the treated cells was collected and stored at −80 °C until use. Stored CM was used to measure the secreted NGF levels using commercial ELISA kits. Secreted NGF levels were determined using the protocol provided by the manufacturer (R&D Systems).

### 4.7. Statistical Analysis

The results are expressed as the mean ± standard error of the mean (SEM). One-way analysis of variance followed by Tukey’s post-hoc test was used to measure statistical significance using GraphPad Prism 5 (La Jolla, CA, USA). Statistical significance was set at *p* < 0.05.

## Data Availability

The data presented in this study are available on request from the corresponding author.

## Supplementary Materials

The following supporting information can be downloaded at: https://www.mdpi.com/article/10.3390/molecules29133037/s1, Figure S1: Effects of isoflavonoids and homoisoflavonoid SH66 on NO production in LPS-primed BV2 microglia cells. Cells were pre-treated with isoflavonoids including daidzin, daidzein, genestin, genestein, and equol, or homoisoflavonoid SH66 followed by LPS (100 ng/mL) activation and incubated overnight. Nitric oxide (NO) production was measured in CM using the Griess reagent assay.
