# Correction: Samsuzzaman et al. A Synthetic Derivative SH 66 of Homoisoflavonoid from Liliaceae Exhibits Anti-Neuroinflammatory Activity against LPS-Induced Microglial Cells. *Molecules* 2024, *29*, 3037

**DOI:** 10.3390/molecules29163926

**Published:** 2024-08-20

**Authors:** Md Samsuzzaman, Lalita Subedi, Seong-Min Hong, Sanha Lee, Bhakta Prasad Gaire, Eun-Ji Ko, Ji-Woong Choi, Seung-Yong Seo, Sun-Yeou Kim

**Affiliations:** 1 College of Pharmacy and Gachon Institute of Pharmaceutical Sciences, Gachon University, Incheon 21936, Republic of Korea; samsuzzaman238@gmail.com (M.S.); subedilali@gmail.com (L.S.); hongsm0517@gmail.com (S.-M.H.); sanha030@gmail.com (S.L.); samarpanbp@gmail.com (B.P.G.); imko1004@gachon.ac.kr (E.-J.K.); pharmchoi@gachon.ac.kr (J.-W.C.); 2 Department of Biochemistry and Molecular Biology, University of Maryland, Baltimore, MD 21201, USA

**Error in Figure**

In the original publication [1], there was a mistake in the Figure 4 image file. Upon reviewing our published work, we realized that the results of the positive control as L-NMMA (PC) on the protein level of the NRLP3 inflammasome in LPS-induced BV2 microglia cells in Figure 4A in Section 2.4 were found to be mishandled in the manuscript preparation. The PC group was also co-treated with LPS in microglia cells. Thus, the Western blotting image in the LPS section from the PC group was changed from “−“to “+”. There was also a mistake in the legend for Figure 4. We missed the Figure 4 legend, which related to a significant difference with the untreated control group. The correct Figure 4 image and legend appear below.

**Figure 4:**
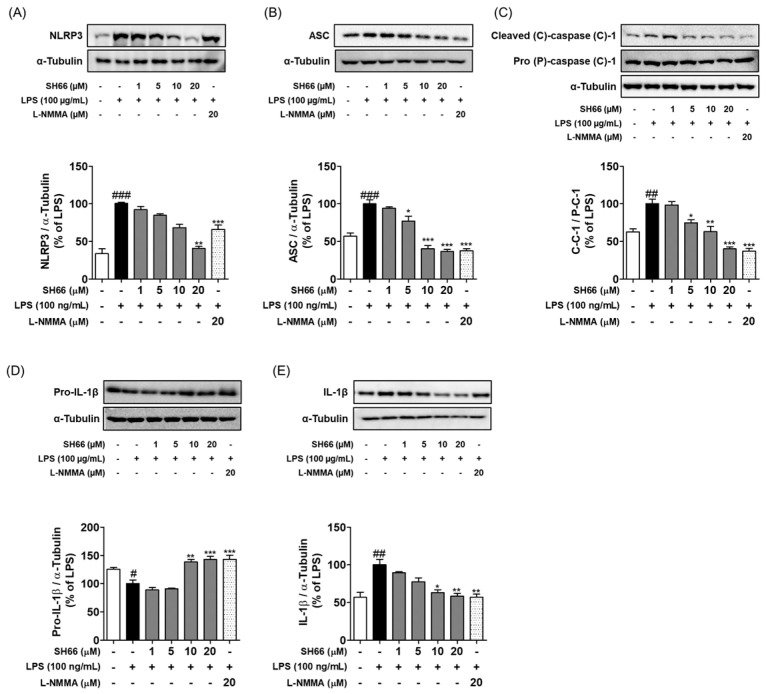
SH66 inhibits NLRP3 inflammasome induction and activation in LPS-primed microglial cells. BV2 cells were pre-treated with SH66 (1–20 μM) followed by the priming of LPS (100 ng/mL) and incubated for 6 h. Protein level was analyzed by Western blot analysis. (**A**–**E**) Protein levels and their quantitative analysis for NLRP3, ASC, pro-caspase-1, cleaved-caspase-1, pro- IL-1β, and IL-1β. α-tubulin was used as loading control. The data shown represent the mean ± SEM (*n* = 3). * *p* < 0.05, ** *p* < 0.01, and *** *p* < 0.001 vs. LPS alone. # *p* < 0.05, ## *p* < 0.01, and ### *p* < 0.001 vs. untreated control group.

The authors state that the scientific conclusions are unaffected. This correction was approved by the Academic Editor. The original publication has also been updated.
