# Supplementary material for: A Synthetic Derivative SH 66 of Homoisoflavonoid from Liliaceae Exhibits Anti-Neuroinflammatory Activity against LPS-Induced Microglial Cells
Source: Molecules. 2024 Jun 26;29(13):3037. doi: 10.3390/molecules29133037 (PMC11243437; doi:10.3390/molecules29133037)
Supplement: Supplementary file 1 [file molecules-29-03037-s001.zip › molecules-3049630-supplementary.pdf]

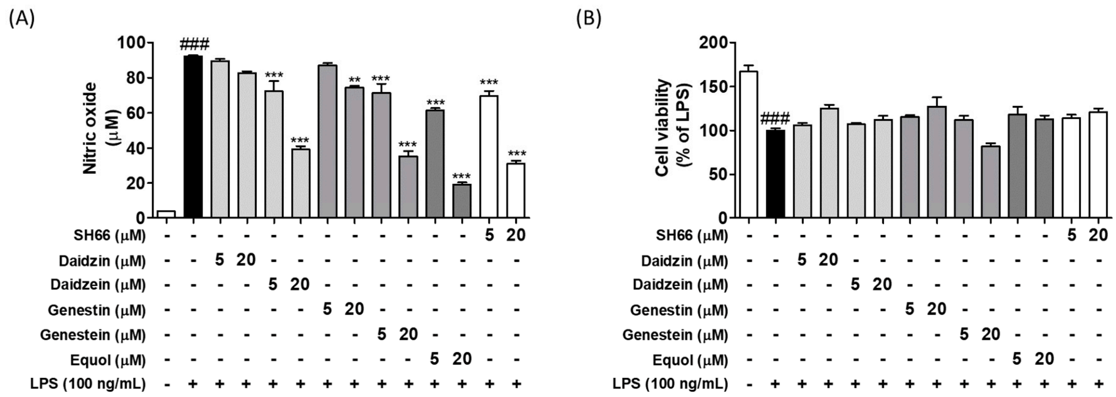

Figure S1. Effects of isoflavonoids and homoisoflavonoid SH66 on NO production in LPS-primed BV2 microglia cells. Cells were pre-treated with isoflavonoids including daidzin, daidzein, genestin, genestein, and equol, or homoisoflavonoid SH66 followed by LPS (100 ng/mL) activation and incubated overnight. Nitric oxide (NO) production was measured in CM using the Griess reagent assay.
